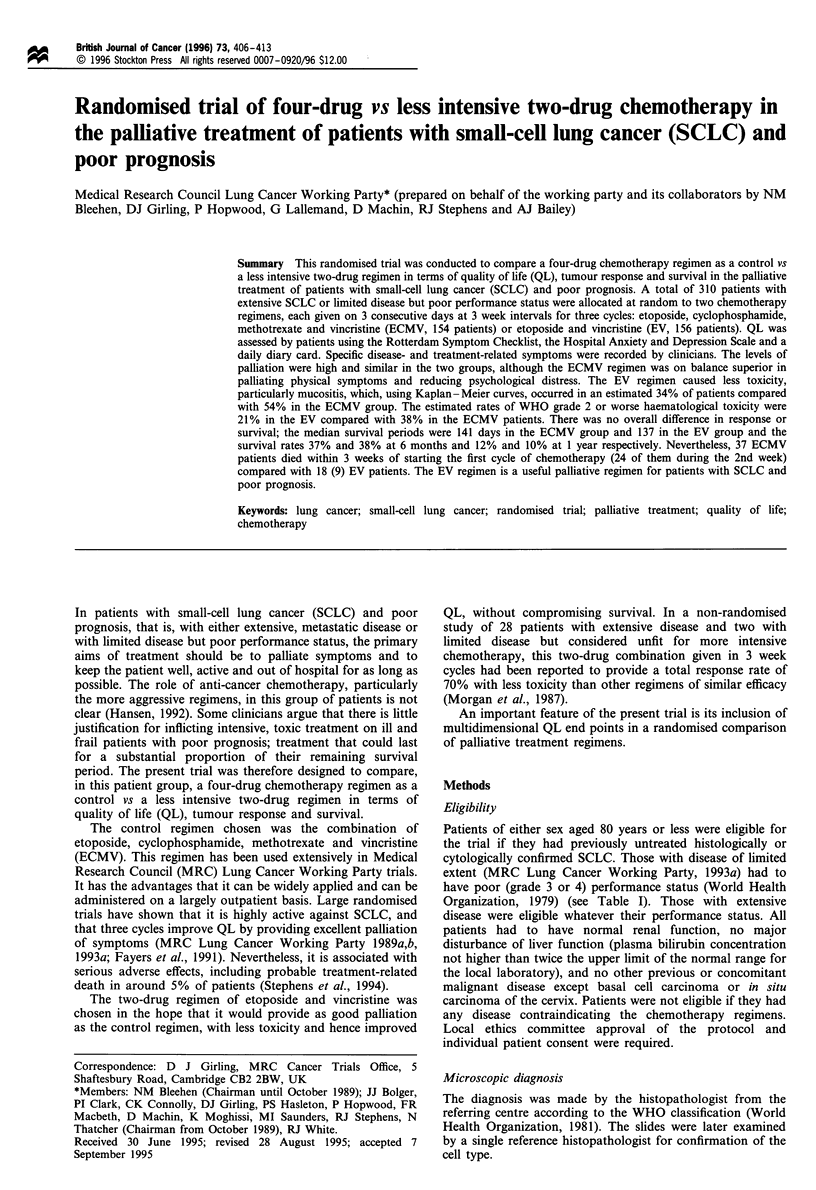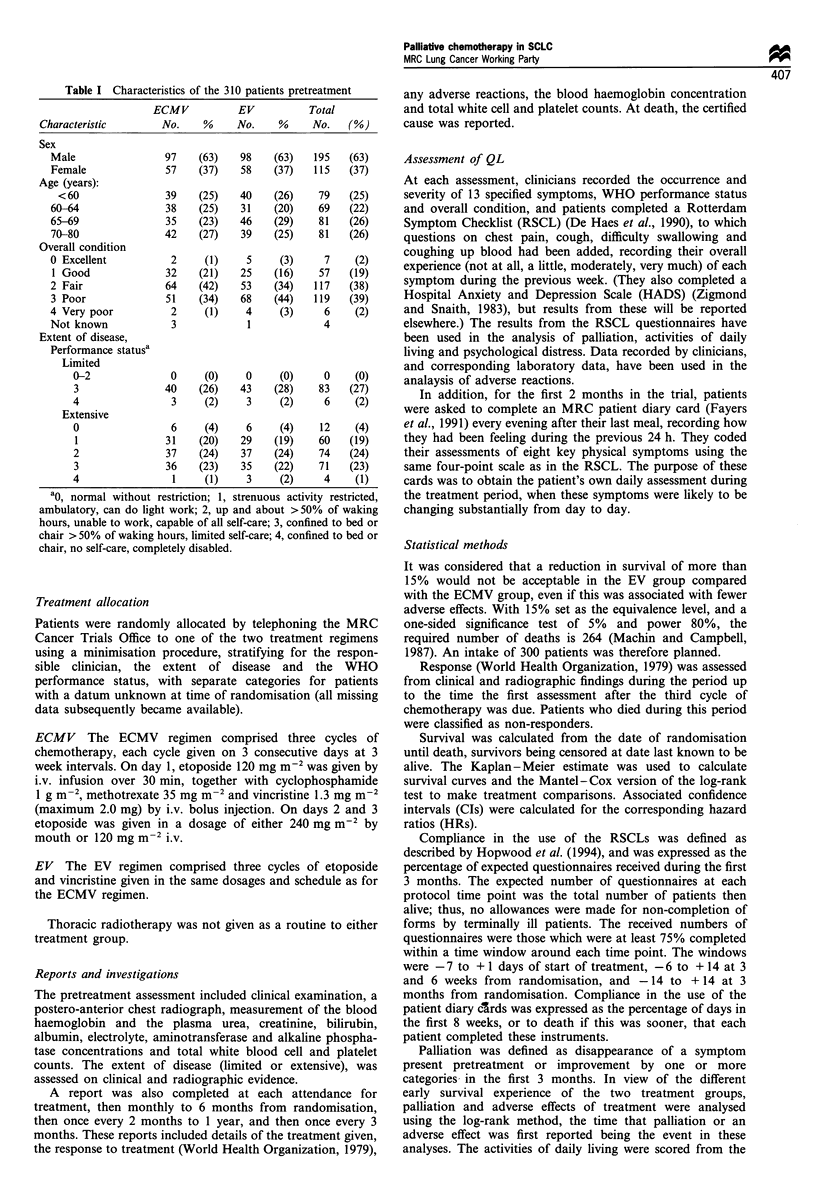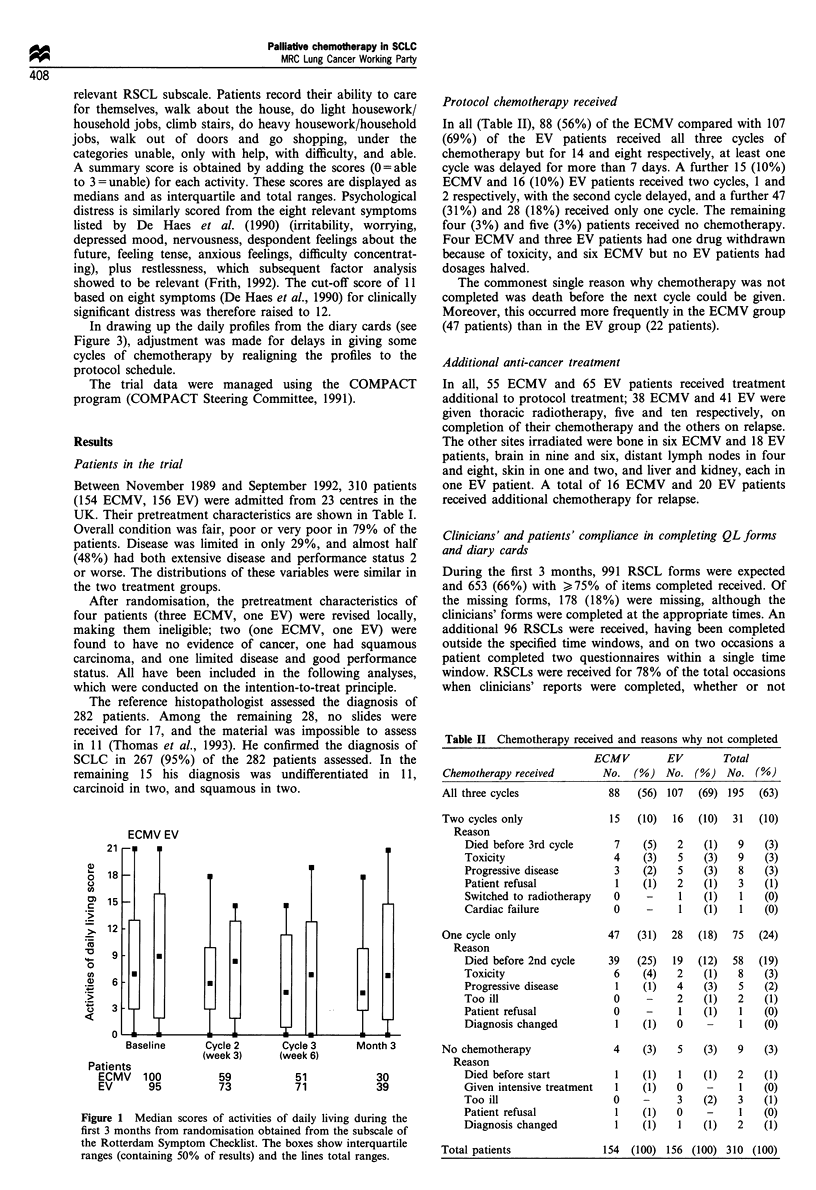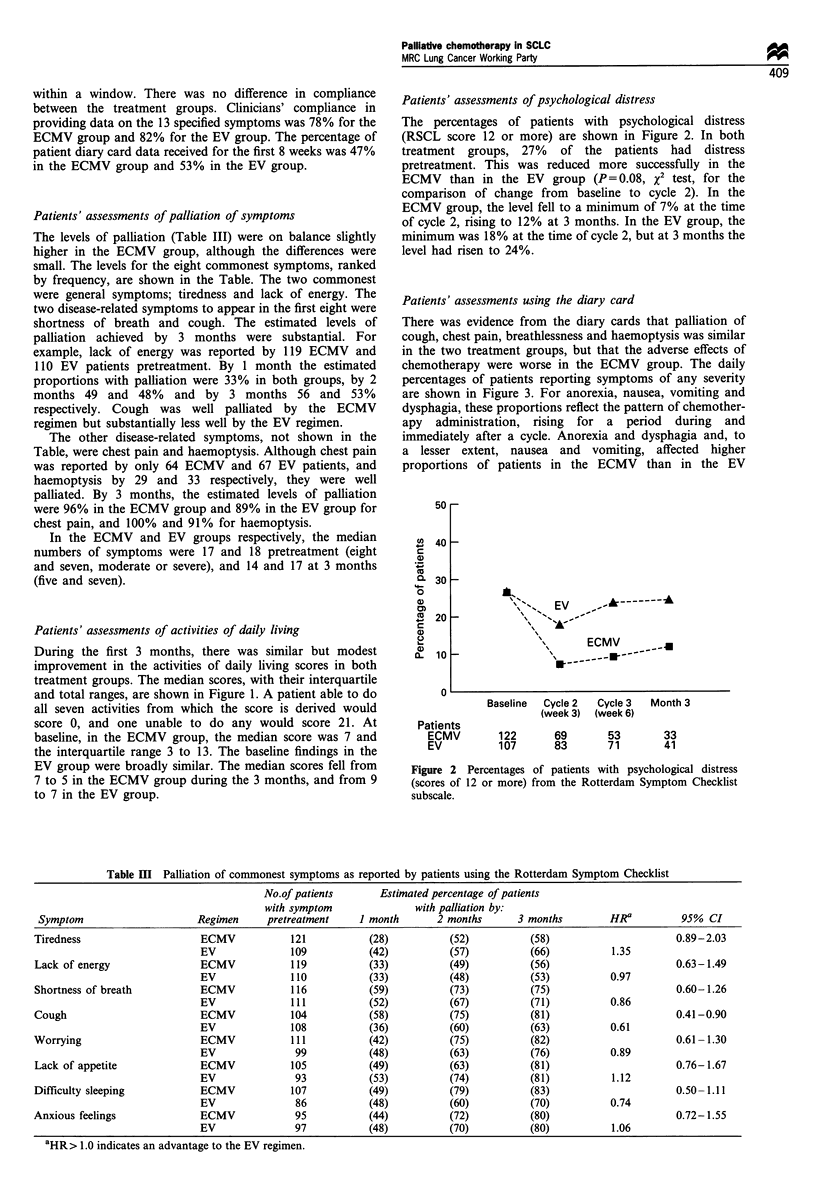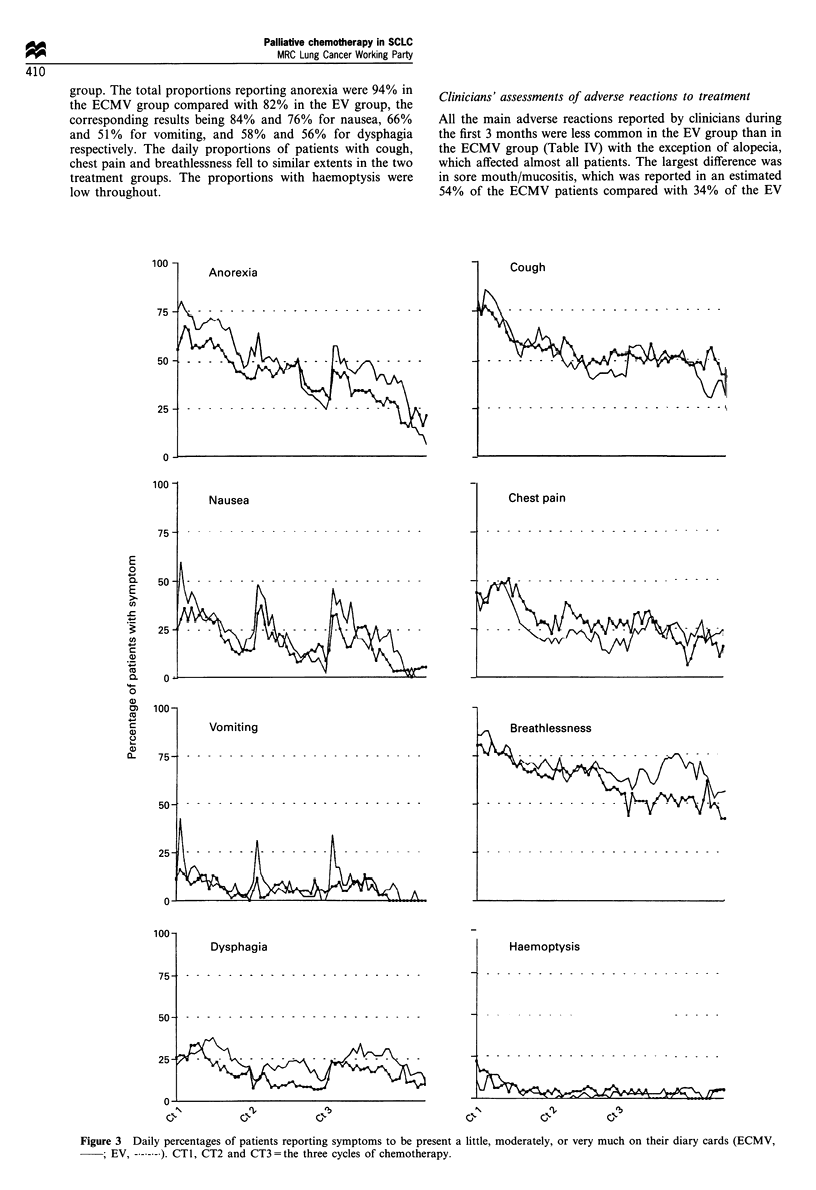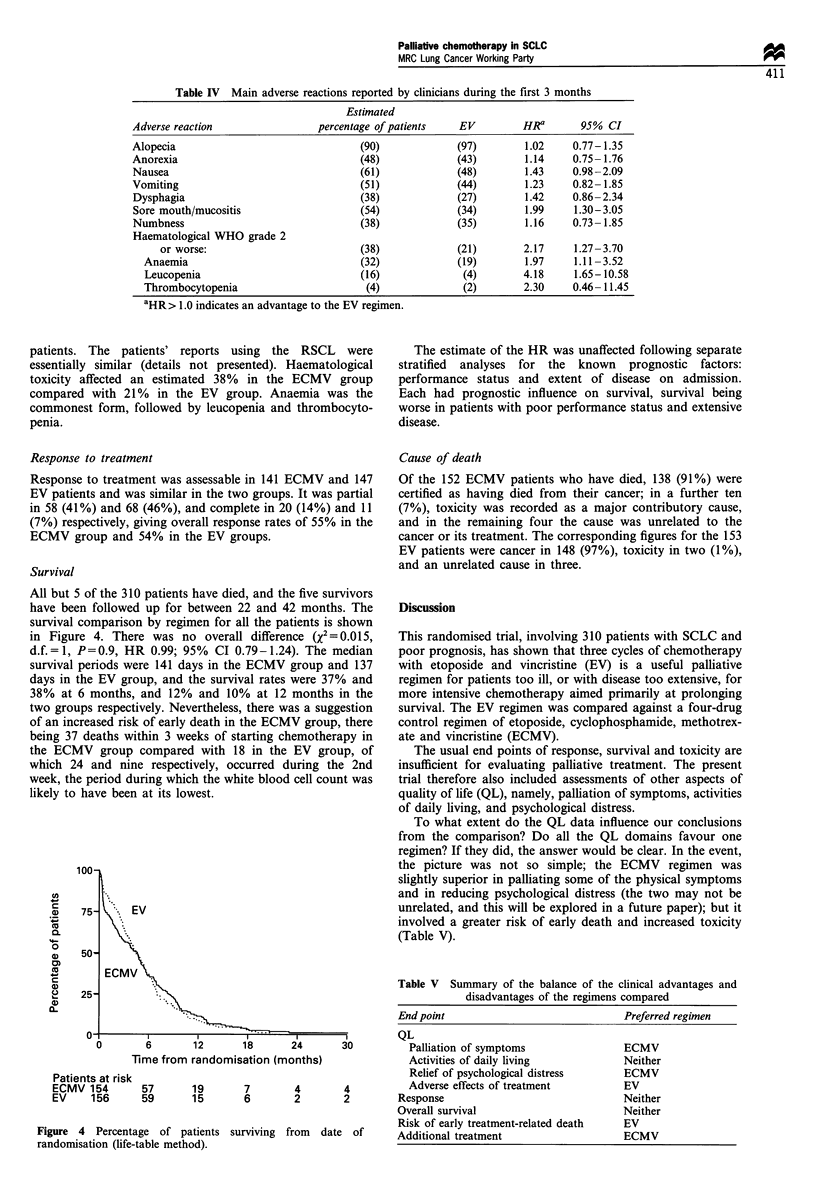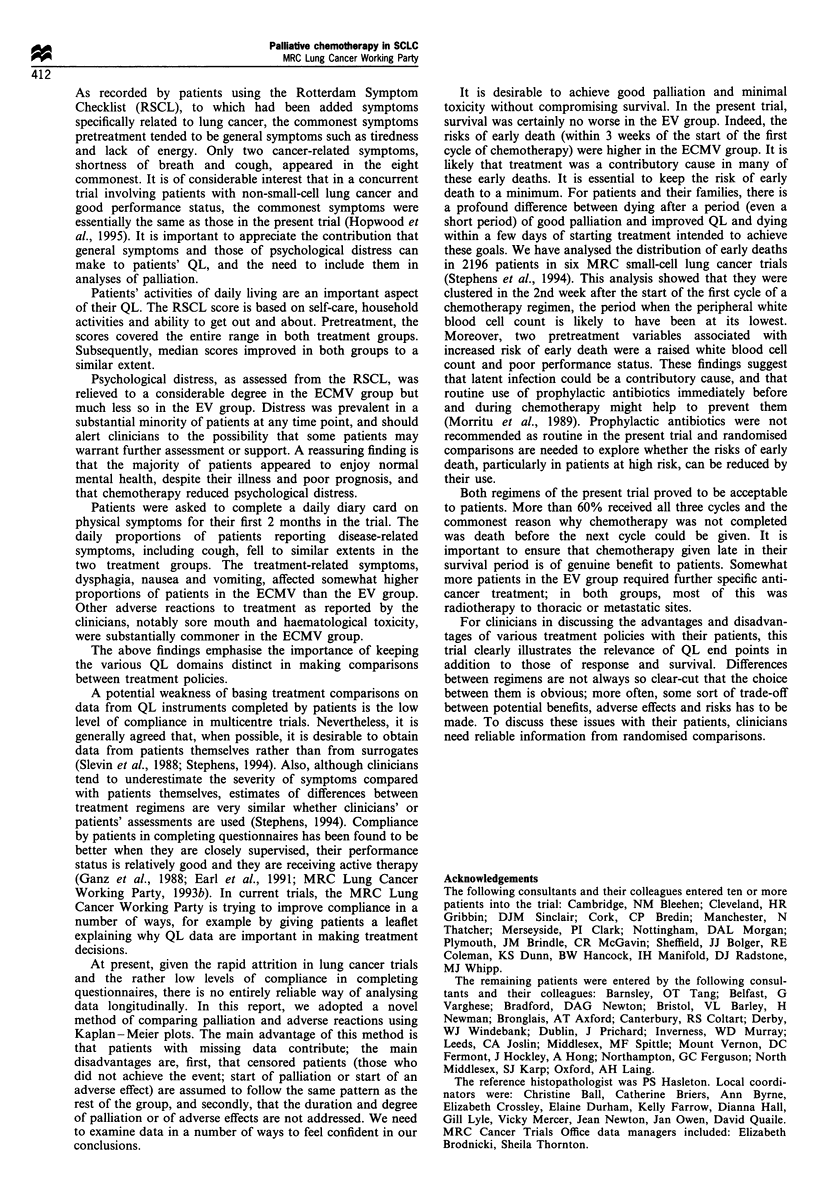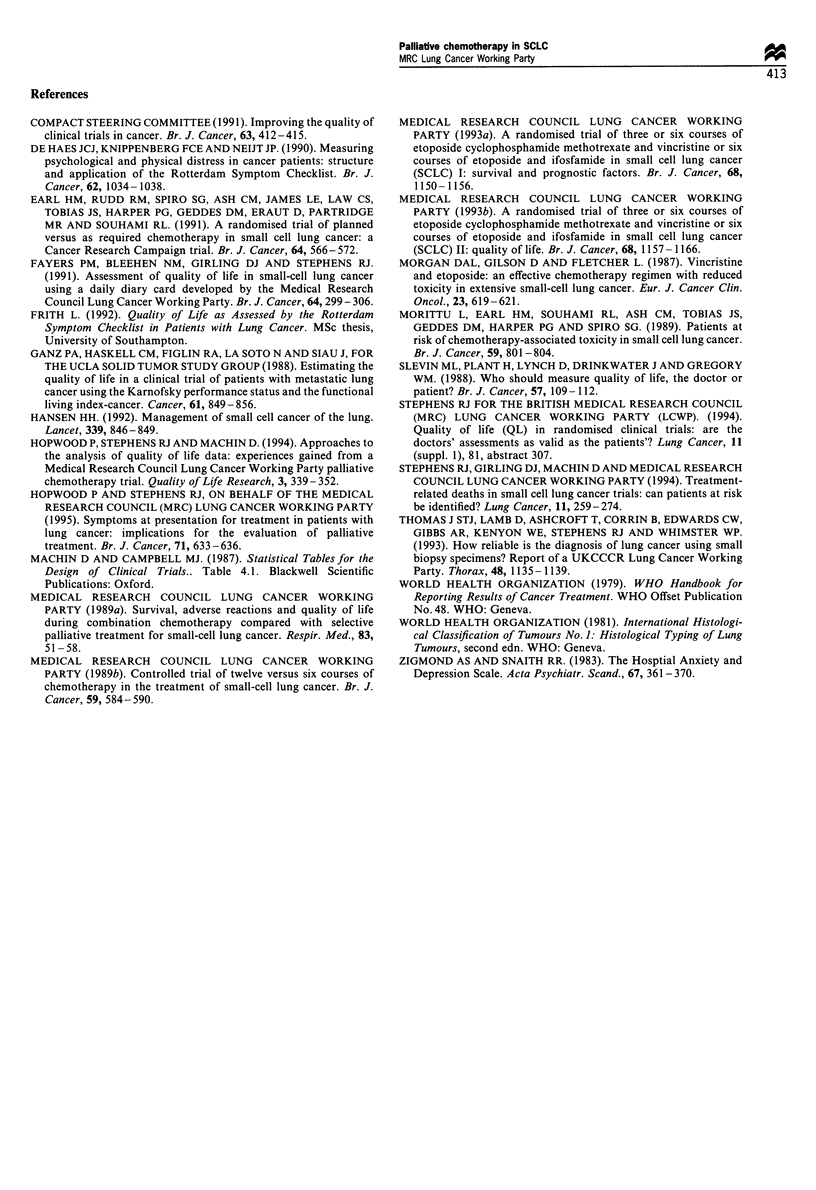# Randomised trial of four-drug vs less intensive two-drug chemotherapy in the palliative treatment of patients with small-cell lung cancer (SCLC) and poor prognosis. Medical Research Council Lung Cancer Working Party.

**DOI:** 10.1038/bjc.1996.71

**Published:** 1996-02

**Authors:** 

## Abstract

This randomised trial was conducted to compare a four-drug chemotherapy regimen as a control vs a less intensive two-drug regimen in terms of quality of life (QL), tumour response and survival in the palliative treatment of patients with small-cell lung cancer (SCLC) and poor prognosis. A total of 310 patients with extensive SCLC or limited disease but poor performance status were allocated at random to two chemotherapy regimens, each given on 3 consecutive days at 3 week intervals for three cycles: etoposide, cyclophosphamide, methotrexate and vincristine (ECMV, 154 patients) or etoposide and vincristine (EV, 156 patients). QL was assessed by patients using the Rotterdam Symptom Checklist, the Hospital Anxiety and Depression Scale and a daily diary card. Specific disease- and treatment-related symptoms were recorded by clinicians. The levels of palliation were high and similar in the two groups, although the ECMV regimen was on balance superior in palliating physical symptoms and reducing psychological distress. The EV regimen caused less toxicity, particularly mucositis, which, using Kaplan-Meier curves, occurred in an estimated 34% of patients compared with 54% in the ECMV group. The estimated rates of WHO grade 2 or worse haematological toxicity were 21% in the EV compared with 38% in the ECMV patients. There was no overall difference in response or survival; the median survival periods were 141 days in the ECMV group and 137 in the EV group and the survival rates 37% and 38% at 6 months and 12% and 10% at 1 year respectively. Nevertheless, 37 ECMV patients died within 3 weeks of starting the first cycle of chemotherapy (24 of them during the 2nd week) compared with 18 (9) EV patients. The EV regimen is a useful palliative regimen for patients with SCLC and poor prognosis.


British Journal of Cancer (1996) 73, 406-413
© 1996 Stockton Press All rights reserved 0007-0920/96 $12.00

### Randomised trial of four-drug *vs* less intensive two-drug chemotherapy in the palliative treatment of patients with small-cell lung cancer (SCLC) and poor prognosis

Medical Research Council Lung Cancer Working Party* (prepared on behalf of the working party and its collaborators by NM Bleehen, DJ Girling, P Hopwood, G Lallemand, D Machin, RJ Stephens and AJ Bailey)

**Summary** This randomised trial was conducted to compare a four-drug chemotherapy regimen as a control *vs* a less intensive two-drug regimen in terms of quality of life (QL), tumour response and survival in the palliative treatment of patients with small-cell lung cancer (SCLC) and poor prognosis. A total of 310 patients with extensive SCLC or limited disease but poor performance status were allocated at random to two chemotherapy regimens, each given on 3 consecutive days at 3 week intervals for three cycles: etoposide, cyclophosphamide, methotrexate and vincristine (ECMV, 154 patients) or etoposide and vincristine (EV, 156 patients). QL was assessed by patients using the Rotterdam Symptom Checklist, the Hospital Anxiety and Depression Scale and a daily diary card. Specific disease- and treatment-related symptoms were recorded by clinicians. The levels of palliation were high and similar in the two groups, although the ECMV regimen was on balance superior in palliating physical symptoms and reducing psychological distress. The EV regimen caused less toxicity, particularly mucositis, which, using Kaplan–Meier curves, occurred in an estimated 34% of patients compared with 54% in the ECMV group. The estimated rates of WHO grade 2 or worse haematological toxicity were 21% in the EV compared with 38% in the ECMV patients. There was no overall difference in response or survival; the median survival periods were 141 days in the ECMV group and 137 in the EV group and the survival rates 37% and 38% at 6 months and 12% and 10% at 1 year respectively. Nevertheless, 37 ECMV patients died within 3 weeks of starting the first cycle of chemotherapy (24 of them during the 2nd week) compared with 18 (9) EV patients. The EV regimen is a useful palliative regimen for patients with SCLC and poor prognosis.

**Keywords:** lung cancer; small-cell lung cancer; randomised trial; palliative treatment; quality of life; chemotherapy

In patients with small-cell lung cancer (SCLC) and poor prognosis, that is, with either extensive, metastatic disease or with limited disease but poor performance status, the primary aims of treatment should be to palliate symptoms and to keep the patient well, active and out of hospital for as long as possible. The role of anti-cancer chemotherapy, particularly the more aggressive regimens, in this group of patients is not clear (Hansen, 1992). Some clinicians argue that there is little justification for inflicting intensive, toxic treatment on ill and frail patients with poor prognosis; treatment that could last for a substantial proportion of their remaining survival period. The present trial was therefore designed to compare, in this patient group, a four-drug chemotherapy regimen as a control *vs* a less intensive two-drug regimen in terms of quality of life (QL), tumour response and survival.

The control regimen chosen was the combination of etoposide, cyclophosphamide, methotrexate and vincristine (ECMV). This regimen has been used extensively in Medical Research Council (MRC) Lung Cancer Working Party trials. It has the advantages that it can be widely applied and can be administered on a largely outpatient basis. Large randomised trials have shown that it is highly active against SCLC, and that three cycles improve QL by providing excellent palliation of symptoms (MRC Lung Cancer Working Party 1989*a*,*b*, 1993*a*; Fayers *et al.*, 1991). Nevertheless, it is associated with serious adverse effects, including probable treatment-related death in around 5% of patients (Stephens *et al.*, 1994).

The two-drug regimen of etoposide and vincristine was chosen in the hope that it would provide as good palliation as the control regimen, with less toxicity and hence improved QL, without compromising survival. In a non-randomised study of 28 patients with extensive disease and two with limited disease but considered unfit for more intensive chemotherapy, this two-drug combination given in 3 week cycles had been reported to provide a total response rate of 70% with less toxicity than other regimens of similar efficacy (Morgan *et al.*, 1987).

An important feature of the present trial is its inclusion of multidimensional QL end points in a randomised comparison of palliative treatment regimens.

### Methods

### *Eligibility*

Patients of either sex aged 80 years or less were eligible for the trial if they had previously untreated histologically or cytologically confirmed SCLC. Those with disease of limited extent (MRC Lung Cancer Working Party, 1993*a*) had to have poor (grade 3 or 4) performance status (World Health Organization, 1979) (see Table I). Those with extensive disease were eligible whatever their performance status. All patients had to have normal renal function, no major disturbance of liver function (plasma bilirubin concentration not higher than twice the upper limit of the normal range for the local laboratory), and no other previous or concomitant malignant disease except basal cell carcinoma or *in situ* carcinoma of the cervix. Patients were not eligible if they had any disease contraindicating the chemotherapy regimens. Local ethics committee approval of the protocol and individual patient consent were required.

### *Microscopic diagnosis*

The diagnosis was made by the histopathologist from the referring centre according to the WHO classification (World Health Organization, 1981). The slides were later examined by a single reference histopathologist for confirmation of the cell type.

Correspondence: D J Girling, MRC Cancer Trials Office, 5 Shaftesbury Road, Cambridge CB2 2BW, UK
*Members: NM Bleehen (Chairman until October 1989); JJ Bolger, PI Clark, CK Connolly, DJ Girling, PS Hasleton, P Hopwood, FR Macbeth, D Machin, K Moghissi, MI Saunders, RJ Stephens, N Thatcher (Chairman from October 1989), RJ White.
Received 30 June 1995; revised 28 August 1995; accepted 7 September 1995

**Table I** Characteristics of the 310 patients pretreatment

| Characteristic | *ECMV* No. | % | *EV* No. | % | *Total* No. | (%) |
|---|---|---|---|---|---|---|
| Sex | | | | | | |
| Male | 97 | (63) | 98 | (63) | 195 | (63) |
| Female | 57 | (37) | 58 | (37) | 115 | (37) |
| Age (years): | | | | | | |
| <60 | 39 | (25) | 40 | (26) | 79 | (25) |
| 60–64 | 38 | (25) | 31 | (20) | 69 | (22) |
| 65–69 | 35 | (23) | 46 | (29) | 81 | (26) |
| 70–80 | 42 | (27) | 39 | (25) | 81 | (26) |
| Overall condition | | | | | | |
| 0 Excellent | 2 | (1) | 5 | (3) | 7 | (2) |
| 1 Good | 32 | (21) | 25 | (16) | 57 | (19) |
| 2 Fair | 64 | (42) | 53 | (34) | 117 | (38) |
| 3 Poor | 51 | (34) | 68 | (44) | 119 | (39) |
| 4 Very poor | 2 | (1) | 4 | (3) | 6 | (2) |
| Not known | 3 | | 1 | | 4 | |
| Extent of disease, Performance status[a] | | | | | | |
| Limited | | | | | | |
| 0–2 | 0 | (0) | 0 | (0) | 0 | (0) |
| 3 | 40 | (26) | 43 | (28) | 83 | (27) |
| 4 | 3 | (2) | 3 | (2) | 6 | (2) |
| Extensive | | | | | | |
| 0 | 6 | (4) | 6 | (4) | 12 | (4) |
| 1 | 31 | (20) | 29 | (19) | 60 | (19) |
| 2 | 37 | (24) | 37 | (24) | 74 | (24) |
| 3 | 36 | (23) | 35 | (22) | 71 | (23) |
| 4 | 1 | (1) | 3 | (2) | 4 | (1) |

[a]0, normal without restriction; 1, strenuous activity restricted, ambulatory, can do light work; 2, up and about >50% of waking hours, unable to work, capable of all self-care; 3, confined to bed or chair >50% of waking hours, limited self-care; 4, confined to bed or chair, no self-care, completely disabled.

### *Treatment allocation*

Patients were randomly allocated by telephoning the MRC Cancer Trials Office to one of the two treatment regimens using a minimisation procedure, stratifying for the responsible clinician, the extent of disease and the WHO performance status, with separate categories for patients with a datum unknown at time of randomisation (all missing data subsequently became available).

*ECMV* The ECMV regimen comprised three cycles of chemotherapy, each cycle given on 3 consecutive days at 3 week intervals. On day 1, etoposide 120 mg $m^{-2}$ was given by i.v. infusion over 30 min, together with cyclophosphamide 1 g $m^{-2}$, methotrexate 35 mg $m^{-2}$ and vincristine 1.3 mg $m^{-2}$ (maximum 2.0 mg) by i.v. bolus injection. On days 2 and 3 etoposide was given in a dosage of either 240 mg $m^{-2}$ by mouth or 120 mg $m^{-2}$ i.v.

*EV* The EV regimen comprised three cycles of etoposide and vincristine given in the same dosages and schedule as for the ECMV regimen.

Thoracic radiotherapy was not given as a routine to either treatment group.

### *Reports and investigations*

The pretreatment assessment included clinical examination, a postero-anterior chest radiograph, measurement of the blood haemoglobin and the plasma urea, creatinine, bilirubin, albumin, electrolyte, aminotransferase and alkaline phosphatase concentrations and total white blood cell and platelet counts. The extent of disease (limited or extensive), was assessed on clinical and radiographic evidence.

A report was also completed at each attendance for treatment, then monthly to 6 months from randomisation, then once every 2 months to 1 year, and then once every 3 months. These reports included details of the treatment given, the response to treatment (World Health Organization, 1979), any adverse reactions, the blood haemoglobin concentration and total white cell and platelet counts. At death, the certified cause was reported.

### *Assessment of QL*

At each assessment, clinicians recorded the occurrence and severity of 13 specified symptoms, WHO performance status and overall condition, and patients completed a Rotterdam Symptom Checklist (RSCL) (De Haes *et al.*, 1990), to which questions on chest pain, cough, difficulty swallowing and coughing up blood had been added, recording their overall experience (not at all, a little, moderately, very much) of each symptom during the previous week. (They also completed a Hospital Anxiety and Depression Scale (HADS) (Zigmond and Snaith, 1983), but results from these will be reported elsewhere.) The results from the RSCL questionnaires have been used in the analysis of palliation, activities of daily living and psychological distress. Data recorded by clinicians, and corresponding laboratory data, have been used in the analaysis of adverse reactions.

In addition, for the first 2 months in the trial, patients were asked to complete an MRC patient diary card (Fayers *et al.*, 1991) every evening after their last meal, recording how they had been feeling during the previous 24 h. They coded their assessments of eight key physical symptoms using the same four-point scale as in the RSCL. The purpose of these cards was to obtain the patient's own daily assessment during the treatment period, when these symptoms were likely to be changing substantially from day to day.

### *Statistical methods*

It was considered that a reduction in survival of more than 15% would not be acceptable in the EV group compared with the ECMV group, even if this was associated with fewer adverse effects. With 15% set as the equivalence level, and a one-sided significance test of 5% and power 80%, the required number of deaths is 264 (Machin and Campbell, 1987). An intake of 300 patients was therefore planned.

Response (World Health Organization, 1979) was assessed from clinical and radiographic findings during the period up to the time the first assessment after the third cycle of chemotherapy was due. Patients who died during this period were classified as non-responders.

Survival was calculated from the date of randomisation until death, survivors being censored at date last known to be alive. The Kaplan–Meier estimate was used to calculate survival curves and the Mantel–Cox version of the log-rank test to make treatment comparisons. Associated confidence intervals (CIs) were calculated for the corresponding hazard ratios (HRs).

Compliance in the use of the RSCLs was defined as described by Hopwood *et al.* (1994), and was expressed as the percentage of expected questionnaires received during the first 3 months. The expected number of questionnaires at each protocol time point was the total number of patients then alive; thus, no allowances were made for non-completion of forms by terminally ill patients. The received numbers of questionnaires were those which were at least 75% completed within a time window around each time point. The windows were −7 to +1 days of start of treatment, −6 to +14 at 3 and 6 weeks from randomisation, and −14 to +14 at 3 months from randomisation. Compliance in the use of the patient diary cards was expressed as the percentage of days in the first 8 weeks, or to death if this was sooner, that each patient completed these instruments.

Palliation was defined as disappearance of a symptom present pretreatment or improvement by one or more categories in the first 3 months. In view of the different early survival experience of the two treatment groups, palliation and adverse effects of treatment were analysed using the log-rank method, the time that palliation or an adverse effect was first reported being the event in these analyses. The activities of daily living were scored from the relevant RSCL subscale. Patients record their ability to care for themselves, walk about the house, do light housework/household jobs, climb stairs, do heavy housework/household jobs, walk out of doors and go shopping, under the categories unable, only with help, with difficulty, and able. A summary score is obtained by adding the scores (0 = able to 3 = unable) for each activity. These scores are displayed as medians and as interquartile and total ranges. Psychological distress is similarly scored from the eight relevant symptoms listed by De Haes *et al.* (1990) (irritability, worrying, depressed mood, nervousness, despondent feelings about the future, feeling tense, anxious feelings, difficulty concentrating), plus restlessness, which subsequent factor analysis showed to be relevant (Frith, 1992). The cut-off score of 11 based on eight symptoms (De Haes *et al.*, 1990) for clinically significant distress was therefore raised to 12.

In drawing up the daily profiles from the diary cards (see Figure 3), adjustment was made for delays in giving some cycles of chemotherapy by realigning the profiles to the protocol schedule.

The trial data were managed using the COMPACT program (COMPACT Steering Committee, 1991).

### Results

### *Patients in the trial*

Between November 1989 and September 1992, 310 patients (154 ECMV, 156 EV) were admitted from 23 centres in the UK. Their pretreatment characteristics are shown in Table I. Overall condition was fair, poor or very poor in 79% of the patients. Disease was limited in only 29%, and almost half (48%) had both extensive disease and performance status 2 or worse. The distributions of these variables were similar in the two treatment groups.

After randomisation, the pretreatment characteristics of four patients (three ECMV, one EV) were revised locally, making them ineligible; two (one ECMV, one EV) were found to have no evidence of cancer, one had squamous carcinoma, and one limited disease and good performance status. All have been included in the following analyses, which were conducted on the intention-to-treat principle.

The reference histopathologist assessed the diagnosis of 282 patients. Among the remaining 28, no slides were received for 17, and the material was impossible to assess in 11 (Thomas *et al.*, 1993). He confirmed the diagnosis of SCLC in 267 (95%) of the 282 patients assessed. In the remaining 15 his diagnosis was undifferentiated in 11, carcinoid in two, and squamous in two.

**Figure 1** Median scores of activities of daily living during the first 3 months from randomisation obtained from the subscale of the Rotterdam Symptom Checklist. The boxes show interquartile ranges (containing 50% of results) and the lines total ranges.

### *Protocol chemotherapy received*

In all (Table II), 88 (56%) of the ECMV compared with 107 (69%) of the EV patients received all three cycles of chemotherapy but for 14 and eight respectively, at least one cycle was delayed for more than 7 days. A further 15 (10%) ECMV and 16 (10%) EV patients received two cycles, 1 and 2 respectively, with the second cycle delayed, and a further 47 (31%) and 28 (18%) received only one cycle. The remaining four (3%) and five (3%) patients received no chemotherapy. Four ECMV and three EV patients had one drug withdrawn because of toxicity, and six ECMV but no EV patients had dosages halved.

The commonest single reason why chemotherapy was not completed was death before the next cycle could be given. Moreover, this occurred more frequently in the ECMV group (47 patients) than in the EV group (22 patients).

### *Additional anti-cancer treatment*

In all, 55 ECMV and 65 EV patients received treatment additional to protocol treatment; 38 ECMV and 41 EV were given thoracic radiotherapy, five and ten respectively, on completion of their chemotherapy and the others on relapse. The other sites irradiated were bone in six ECMV and 18 EV patients, brain in nine and six, distant lymph nodes in four and eight, skin in one and two, and liver and kidney, each in one EV patient. A total of 16 ECMV and 20 EV patients received additional chemotherapy for relapse.

### *Clinicians' and patients' compliance in completing QL forms and diary cards*

During the first 3 months, 991 RSCL forms were expected and 653 (66%) with ⩾75% of items completed received. Of the missing forms, 178 (18%) were missing, although the clinicians' forms were completed at the appropriate times. An additional 96 RSCLs were received, having been completed outside the specified time windows, and on two occasions a patient completed two questionnaires within a single time window. RSCLs were received for 78% of the total occasions when clinicians' reports were completed, whether or not

**Table II** Chemotherapy received and reasons why not completed

| *Chemotherapy received* | *ECMV No.* | *(%)* | *EV No.* | *(%)* | *Total No.* | *(%)* |
|---|---|---|---|---|---|---|
| All three cycles | 88 | (56) | 107 | (69) | 195 | (63) |
| Two cycles only | 15 | (10) | 16 | (10) | 31 | (10) |
| Reason | | | | | | |
| Died before 3rd cycle | 7 | (5) | 2 | (1) | 9 | (3) |
| Toxicity | 4 | (3) | 5 | (3) | 9 | (3) |
| Progressive disease | 3 | (2) | 5 | (3) | 8 | (3) |
| Patient refusal | 1 | (1) | 2 | (1) | 3 | (1) |
| Switched to radiotherapy | 0 | – | 1 | (1) | 1 | (0) |
| Cardiac failure | 0 | – | 1 | (1) | 1 | (0) |
| One cycle only | 47 | (31) | 28 | (18) | 75 | (24) |
| Reason | | | | | | |
| Died before 2nd cycle | 39 | (25) | 19 | (12) | 58 | (19) |
| Toxicity | 6 | (4) | 2 | (1) | 8 | (3) |
| Progressive disease | 1 | (1) | 4 | (3) | 5 | (2) |
| Too ill | 0 | – | 2 | (1) | 2 | (1) |
| Patient refusal | 0 | – | 1 | (1) | 1 | (0) |
| Diagnosis changed | 1 | (1) | 0 | – | 1 | (0) |
| No chemotherapy | 4 | (3) | 5 | (3) | 9 | (3) |
| Reason | | | | | | |
| Died before start | 1 | (1) | 1 | (1) | 2 | (1) |
| Given intensive treatment | 1 | (1) | 0 | – | 1 | (0) |
| Too ill | 0 | – | 3 | (2) | 3 | (1) |
| Patient refusal | 1 | (1) | 0 | – | 1 | (0) |
| Diagnosis changed | 1 | (1) | 1 | (1) | 2 | (1) |
| Total patients | 154 | (100) | 156 | (100) | 310 | (100) |

within a window. There was no difference in compliance between the treatment groups. Clinicians' compliance in providing data on the 13 specified symptoms was 78% for the ECMV group and 82% for the EV group. The percentage of patient diary card data received for the first 8 weeks was 47% in the ECMV group and 53% in the EV group.

### *Patients' assessments of palliation of symptoms*

The levels of palliation (Table III) were on balance slightly higher in the ECMV group, although the differences were small. The levels for the eight commonest symptoms, ranked by frequency, are shown in the Table. The two commonest were general symptoms; tiredness and lack of energy. The two disease-related symptoms to appear in the first eight were shortness of breath and cough. The estimated levels of palliation achieved by 3 months were substantial. For example, lack of energy was reported by 119 ECMV and 110 EV patients pretreatment. By 1 month the estimated proportions with palliation were 33% in both groups, by 2 months 49 and 48% and by 3 months 56 and 53% respectively. Cough was well palliated by the ECMV regimen but substantially less well by the EV regimen.

The other disease-related symptoms, not shown in the Table, were chest pain and haemoptysis. Although chest pain was reported by only 64 ECMV and 67 EV patients, and haemoptysis by 29 and 33 respectively, they were well palliated. By 3 months, the estimated levels of palliation were 96% in the ECMV group and 89% in the EV group for chest pain, and 100% and 91% for haemoptysis.

In the ECMV and EV groups respectively, the median numbers of symptoms were 17 and 18 pretreatment (eight and seven, moderate or severe), and 14 and 17 at 3 months (five and seven).

### *Patients' assessments of activities of daily living*

During the first 3 months, there was similar but modest improvement in the activities of daily living scores in both treatment groups. The median scores, with their interquartile and total ranges, are shown in Figure 1. A patient able to do all seven activities from which the score is derived would score 0, and one unable to do any would score 21. At baseline, in the ECMV group, the median score was 7 and the interquartile range 3 to 13. The baseline findings in the EV group were broadly similar. The median scores fell from 7 to 5 in the ECMV group during the 3 months, and from 9 to 7 in the EV group.

### *Patients' assessments of psychological distress*

The percentages of patients with psychological distress (RSCL score 12 or more) are shown in Figure 2. In both treatment groups, 27% of the patients had distress pretreatment. This was reduced more successfully in the ECMV than in the EV group ($P=0.08$, $\chi^2$ test, for the comparison of change from baseline to cycle 2). In the ECMV group, the level fell to a minimum of 7% at the time of cycle 2, rising to 12% at 3 months. In the EV group, the minimum was 18% at the time of cycle 2, but at 3 months the level had risen to 24%.

### *Patients' assessments using the diary card*

There was evidence from the diary cards that palliation of cough, chest pain, breathlessness and haemoptysis was similar in the two treatment groups, but that the adverse effects of chemotherapy were worse in the ECMV group. The daily percentages of patients reporting symptoms of any severity are shown in Figure 3. For anorexia, nausea, vomiting and dysphagia, these proportions reflect the pattern of chemotherapy administration, rising for a period during and immediately after a cycle. Anorexia and dysphagia and, to a lesser extent, nausea and vomiting, affected higher proportions of patients in the ECMV than in the EV

**Figure 2** Percentages of patients with psychological distress (scores of 12 or more) from the Rotterdam Symptom Checklist subscale.

**Table III** Palliation of commonest symptoms as reported by patients using the Rotterdam Symptom Checklist

| *Symptom* | *Regimen* | *No. of patients with symptom pretreatment* | *Estimated percentage of patients with palliation by: 1 month* | *2 months* | *3 months* | *HR[a]* | *95% CI* |
|---|---|---|---|---|---|---|---|
| Tiredness | ECMV | 121 | (28) | (52) | (58) | | 0.89–2.03 |
| | EV | 109 | (42) | (57) | (66) | 1.35 | |
| Lack of energy | ECMV | 119 | (33) | (49) | (56) | | 0.63–1.49 |
| | EV | 110 | (33) | (48) | (53) | 0.97 | |
| Shortness of breath | ECMV | 116 | (59) | (73) | (75) | | 0.60–1.26 |
| | EV | 111 | (52) | (67) | (71) | 0.86 | |
| Cough | ECMV | 104 | (58) | (75) | (81) | | 0.41–0.90 |
| | EV | 108 | (36) | (60) | (63) | 0.61 | |
| Worrying | ECMV | 111 | (42) | (75) | (82) | | 0.61–1.30 |
| | EV | 99 | (48) | (63) | (76) | 0.89 | |
| Lack of appetite | ECMV | 105 | (49) | (63) | (81) | | 0.76–1.67 |
| | EV | 93 | (53) | (74) | (81) | 1.12 | |
| Difficulty sleeping | ECMV | 107 | (49) | (79) | (83) | | 0.50–1.11 |
| | EV | 86 | (48) | (60) | (70) | 0.74 | |
| Anxious feelings | ECMV | 95 | (44) | (72) | (80) | | 0.72–1.55 |
| | EV | 97 | (48) | (70) | (80) | 1.06 | |

[a]HR > 1.0 indicates an advantage to the EV regimen.

group. The total proportions reporting anorexia were 94% in the ECMV group compared with 82% in the EV group, the corresponding results being 84% and 76% for nausea, 66% and 51% for vomiting, and 58% and 56% for dysphagia respectively. The daily proportions of patients with cough, chest pain and breathlessness fell to similar extents in the two treatment groups. The proportions with haemoptysis were low throughout.

*Clinicians' assessments of adverse reactions to treatment*

All the main adverse reactions reported by clinicians during the first 3 months were less common in the EV group than in the ECMV group (Table IV) with the exception of alopecia, which affected almost all patients. The largest difference was in sore mouth/mucositis, which was reported in an estimated 54% of the ECMV patients compared with 34% of the EV

**Figure 3** Daily percentages of patients reporting symptoms to be present a little, moderately, or very much on their diary cards (ECMV, ——; EV, -•-•-•-). CT1, CT2 and CT3 = the three cycles of chemotherapy.

**Table IV** Main adverse reactions reported by clinicians during the first 3 months

| *Adverse reaction* | *Estimated percentage of patients* | *EV* | *HR[a]* | *95% CI* |
|---|---|---|---|---|
| Alopecia | (90) | (97) | 1.02 | 0.77–1.35 |
| Anorexia | (48) | (43) | 1.14 | 0.75–1.76 |
| Nausea | (61) | (48) | 1.43 | 0.98–2.09 |
| Vomiting | (51) | (44) | 1.23 | 0.82–1.85 |
| Dysphagia | (38) | (27) | 1.42 | 0.86–2.34 |
| Sore mouth/mucositis | (54) | (34) | 1.99 | 1.30–3.05 |
| Numbness | (38) | (35) | 1.16 | 0.73–1.85 |
| Haematological WHO grade 2 or worse: | (38) | (21) | 2.17 | 1.27–3.70 |
| Anaemia | (32) | (19) | 1.97 | 1.11–3.52 |
| Leucopenia | (16) | (4) | 4.18 | 1.65–10.58 |
| Thrombocytopenia | (4) | (2) | 2.30 | 0.46–11.45 |

[a]HR > 1.0 indicates an advantage to the EV regimen.

patients. The patients' reports using the RSCL were essentially similar (details not presented). Haematological toxicity affected an estimated 38% in the ECMV group compared with 21% in the EV group. Anaemia was the commonest form, followed by leucopenia and thrombocytopenia.

*Response to treatment*

Response to treatment was assessable in 141 ECMV and 147 EV patients and was similar in the two groups. It was partial in 58 (41%) and 68 (46%), and complete in 20 (14%) and 11 (7%) respectively, giving overall response rates of 55% in the ECMV group and 54% in the EV groups.

*Survival*

All but 5 of the 310 patients have died, and the five survivors have been followed up for between 22 and 42 months. The survival comparison by regimen for all the patients is shown in Figure 4. There was no overall difference ($\chi^2 = 0.015$, d.f. = 1, $P = 0.9$, HR 0.99; 95% CI 0.79–1.24). The median survival periods were 141 days in the ECMV group and 137 days in the EV group, and the survival rates were 37% and 38% at 6 months, and 12% and 10% at 12 months in the two groups respectively. Nevertheless, there was a suggestion of an increased risk of early death in the ECMV group, there being 37 deaths within 3 weeks of starting chemotherapy in the ECMV group compared with 18 in the EV group, of which 24 and nine respectively, occurred during the 2nd week, the period during which the white blood cell count was likely to have been at its lowest.

Patients at risk

| | | | | | | |
|---|---|---|---|---|---|---|
| ECMV | 154 | 57 | 19 | 7 | 4 | 4 |
| EV | 156 | 59 | 15 | 6 | 2 | 2 |

**Figure 4** Percentage of patients surviving from date of randomisation (life-table method).

The estimate of the HR was unaffected following separate stratified analyses for the known prognostic factors: performance status and extent of disease on admission. Each had prognostic influence on survival, survival being worse in patients with poor performance status and extensive disease.

*Cause of death*

Of the 152 ECMV patients who have died, 138 (91%) were certified as having died from their cancer; in a further ten (7%), toxicity was recorded as a major contributory cause, and in the remaining four the cause was unrelated to the cancer or its treatment. The corresponding figures for the 153 EV patients were cancer in 148 (97%), toxicity in two (1%), and an unrelated cause in three.

### Discussion

This randomised trial, involving 310 patients with SCLC and poor prognosis, has shown that three cycles of chemotherapy with etoposide and vincristine (EV) is a useful palliative regimen for patients too ill, or with disease too extensive, for more intensive chemotherapy aimed primarily at prolonging survival. The EV regimen was compared against a four-drug control regimen of etoposide, cyclophosphamide, methotrexate and vincristine (ECMV).

The usual end points of response, survival and toxicity are insufficient for evaluating palliative treatment. The present trial therefore also included assessments of other aspects of quality of life (QL), namely, palliation of symptoms, activities of daily living, and psychological distress.

To what extent do the QL data influence our conclusions from the comparison? Do all the QL domains favour one regimen? If they did, the answer would be clear. In the event, the picture was not so simple; the ECMV regimen was slightly superior in palliating some of the physical symptoms and in reducing psychological distress (the two may not be unrelated, and this will be explored in a future paper); but it involved a greater risk of early death and increased toxicity (Table V).

**Table V** Summary of the balance of the clinical advantages and disadvantages of the regimens compared

| *End point* | *Preferred regimen* |
|---|---|
| QL | |
| Palliation of symptoms | ECMV |
| Activities of daily living | Neither |
| Relief of psychological distress | ECMV |
| Adverse effects of treatment | EV |
| Response | Neither |
| Overall survival | Neither |
| Risk of early treatment-related death | EV |
| Additional treatment | ECMV |

As recorded by patients using the Rotterdam Symptom Checklist (RSCL), to which had been added symptoms specifically related to lung cancer, the commonest symptoms pretreatment tended to be general symptoms such as tiredness and lack of energy. Only two cancer-related symptoms, shortness of breath and cough, appeared in the eight commonest. It is of considerable interest that in a concurrent trial involving patients with non-small-cell lung cancer and good performance status, the commonest symptoms were essentially the same as those in the present trial (Hopwood *et al.*, 1995). It is important to appreciate the contribution that general symptoms and those of psychological distress can make to patients' QL, and the need to include them in analyses of palliation.

Patients' activities of daily living are an important aspect of their QL. The RSCL score is based on self-care, household activities and ability to get out and about. Pretreatment, the scores covered the entire range in both treatment groups. Subsequently, median scores improved in both groups to a similar extent.

Psychological distress, as assessed from the RSCL, was relieved to a considerable degree in the ECMV group but much less so in the EV group. Distress was prevalent in a substantial minority of patients at any time point, and should alert clinicians to the possibility that some patients may warrant further assessment or support. A reassuring finding is that the majority of patients appeared to enjoy normal mental health, despite their illness and poor prognosis, and that chemotherapy reduced psychological distress.

Patients were asked to complete a daily diary card on physical symptoms for their first 2 months in the trial. The daily proportions of patients reporting disease-related symptoms, including cough, fell to similar extents in the two treatment groups. The treatment-related symptoms, dysphagia, nausea and vomiting, affected somewhat higher proportions of patients in the ECMV than the EV group. Other adverse reactions to treatment as reported by the clinicians, notably sore mouth and haematological toxicity, were substantially commoner in the ECMV group.

The above findings emphasise the importance of keeping the various QL domains distinct in making comparisons between treatment policies.

A potential weakness of basing treatment comparisons on data from QL instruments completed by patients is the low level of compliance in multicentre trials. Nevertheless, it is generally agreed that, when possible, it is desirable to obtain data from patients themselves rather than from surrogates (Slevin *et al.*, 1988; Stephens, 1994). Also, although clinicians tend to underestimate the severity of symptoms compared with patients themselves, estimates of differences between treatment regimens are very similar whether clinicians' or patients' assessments are used (Stephens, 1994). Compliance by patients in completing questionnaires has been found to be better when they are closely supervised, their performance status is relatively good and they are receiving active therapy (Ganz *et al.*, 1988; Earl *et al.*, 1991; MRC Lung Cancer Working Party, 1993*b*). In current trials, the MRC Lung Cancer Working Party is trying to improve compliance in a number of ways, for example by giving patients a leaflet explaining why QL data are important in making treatment decisions.

At present, given the rapid attrition in lung cancer trials and the rather low levels of compliance in completing questionnaires, there is no entirely reliable way of analysing data longitudinally. In this report, we adopted a novel method of comparing palliation and adverse reactions using Kaplan–Meier plots. The main advantage of this method is that patients with missing data contribute; the main disadvantages are, first, that censored patients (those who did not achieve the event; start of palliation or start of an adverse effect) are assumed to follow the same pattern as the rest of the group, and secondly, that the duration and degree of palliation or of adverse effects are not addressed. We need to examine data in a number of ways to feel confident in our conclusions.

It is desirable to achieve good palliation and minimal toxicity without compromising survival. In the present trial, survival was certainly no worse in the EV group. Indeed, the risks of early death (within 3 weeks of the start of the first cycle of chemotherapy) were higher in the ECMV group. It is likely that treatment was a contributory cause in many of these early deaths. It is essential to keep the risk of early death to a minimum. For patients and their families, there is a profound difference between dying after a period (even a short period) of good palliation and improved QL and dying within a few days of starting treatment intended to achieve these goals. We have analysed the distribution of early deaths in 2196 patients in six MRC small-cell lung cancer trials (Stephens *et al.*, 1994). This analysis showed that they were clustered in the 2nd week after the start of the first cycle of a chemotherapy regimen, the period when the peripheral white blood cell count is likely to have been at its lowest. Moreover, two pretreatment variables associated with increased risk of early death were a raised white blood cell count and poor performance status. These findings suggest that latent infection could be a contributory cause, and that routine use of prophylactic antibiotics immediately before and during chemotherapy might help to prevent them (Morritu *et al.*, 1989). Prophylactic antibiotics were not recommended as routine in the present trial and randomised comparisons are needed to explore whether the risks of early death, particularly in patients at high risk, can be reduced by their use.

Both regimens of the present trial proved to be acceptable to patients. More than 60% received all three cycles and the commonest reason why chemotherapy was not completed was death before the next cycle could be given. It is important to ensure that chemotherapy given late in their survival period is of genuine benefit to patients. Somewhat more patients in the EV group required further specific anti-cancer treatment; in both groups, most of this was radiotherapy to thoracic or metastatic sites.

For clinicians in discussing the advantages and disadvantages of various treatment policies with their patients, this trial clearly illustrates the relevance of QL end points in addition to those of response and survival. Differences between regimens are not always so clear-cut that the choice between them is obvious; more often, some sort of trade-off between potential benefits, adverse effects and risks has to be made. To discuss these issues with their patients, clinicians need reliable information from randomised comparisons.

**Acknowledgements**
The following consultants and their colleagues entered ten or more patients into the trial: Cambridge, NM Bleehen; Cleveland, HR Gribbin; DJM Sinclair; Cork, CP Bredin; Manchester, N Thatcher; Merseyside, PI Clark; Nottingham, DAL Morgan; Plymouth, JM Brindle, CR McGavin; Sheffield, JJ Bolger, RE Coleman, KS Dunn, BW Hancock, IH Manifold, DJ Radstone, MJ Whipp.

The remaining patients were entered by the following consultants and their colleagues: Barnsley, OT Tang; Belfast, G Varghese; Bradford, DAG Newton; Bristol, VL Barley, H Newman; Bronglais, AT Axford; Canterbury, RS Coltart; Derby, WJ Windebank; Dublin, J Prichard; Inverness, WD Murray; Leeds, CA Joslin; Middlesex, MF Spittle; Mount Vernon, DC Fermont, J Hockley, A Hong; Northampton, GC Ferguson; North Middlesex, SJ Karp; Oxford, AH Laing.

The reference histopathologist was PS Hasleton. Local coordinators were: Christine Ball, Catherine Briers, Ann Byrne, Elizabeth Crossley, Elaine Durham, Kelly Farrow, Dianna Hall, Gill Lyle, Vicky Mercer, Jean Newton, Jan Owen, David Quaile. MRC Cancer Trials Office data managers included: Elizabeth Brodnicki, Sheila Thornton.

### References

COMPACT STEERING COMMITTEE (1991). Improving the quality of clinical trials in cancer. *Br. J. Cancer*, **63**, 412–415.

DE HAES JCJ, KNIPPENBERG FCE AND NEIJT JP. (1990). Measuring psychological and physical distress in cancer patients: structure and application of the Rotterdam Symptom Checklist. *Br. J. Cancer*, **62**, 1034–1038.

EARL HM, RUDD RM, SPIRO SG, ASH CM, JAMES LE, LAW CS, TOBIAS JS, HARPER PG, GEDDES DM, ERAUT D, PARTRIDGE MR AND SOUHAMI RL. (1991). A randomised trial of planned versus as required chemotherapy in small cell lung cancer: a Cancer Research Campaign trial. *Br. J. Cancer*, **64**, 566–572.

FAYERS PM, BLEEHEN NM, GIRLING DJ AND STEPHENS RJ. (1991). Assessment of quality of life in small-cell lung cancer using a daily diary card developed by the Medical Research Council Lung Cancer Working Party. *Br. J. Cancer*, **64**, 299–306.

FRITH L. (1992). *Quality of Life as Assessed by the Rotterdam Symptom Checklist in Patients with Lung Cancer*. MSc thesis, University of Southampton.

GANZ PA, HASKELL CM, FIGLIN RA, LA SOTO N AND SIAU J, FOR THE UCLA SOLID TUMOR STUDY GROUP (1988). Estimating the quality of life in a clinical trial of patients with metastatic lung cancer using the Karnofsky performance status and the functional living index-cancer. *Cancer*, **61**, 849–856.

HANSEN HH. (1992). Management of small cell cancer of the lung. *Lancet*, **339**, 846–849.

HOPWOOD P, STEPHENS RJ AND MACHIN D. (1994). Approaches to the analysis of quality of life data: experiences gained from a Medical Research Council Lung Cancer Working Party palliative chemotherapy trial. *Quality of Life Research*, **3**, 339–352.

HOPWOOD P AND STEPHENS RJ, ON BEHALF OF THE MEDICAL RESEARCH COUNCIL (MRC) LUNG CANCER WORKING PARTY (1995). Symptoms at presentation for treatment in patients with lung cancer: implications for the evaluation of palliative treatment. *Br. J. Cancer*, **71**, 633–636.

MACHIN D AND CAMPBELL MJ. (1987). *Statistical Tables for the Design of Clinical Trials.*. Table 4.1. Blackwell Scientific Publications: Oxford.

MEDICAL RESEARCH COUNCIL LUNG CANCER WORKING PARTY (1989*a*). Survival, adverse reactions and quality of life during combination chemotherapy compared with selective palliative treatment for small-cell lung cancer. *Respir. Med.*, **83**, 51–58.

MEDICAL RESEARCH COUNCIL LUNG CANCER WORKING PARTY (1989*b*). Controlled trial of twelve versus six courses of chemotherapy in the treatment of small-cell lung cancer. *Br. J. Cancer*, **59**, 584–590.

MEDICAL RESEARCH COUNCIL LUNG CANCER WORKING PARTY (1993*a*). A randomised trial of three or six courses of etoposide cyclophosphamide methotrexate and vincristine or six courses of etoposide and ifosfamide in small cell lung cancer (SCLC) I: survival and prognostic factors. *Br. J. Cancer*, **68**, 1150–1156.

MEDICAL RESEARCH COUNCIL LUNG CANCER WORKING PARTY (1993*b*). A randomised trial of three or six courses of etoposide cyclophosphamide methotrexate and vincristine or six courses of etoposide and ifosfamide in small cell lung cancer (SCLC) II: quality of life. *Br. J. Cancer*, **68**, 1157–1166.

MORGAN DAL, GILSON D AND FLETCHER L. (1987). Vincristine and etoposide: an effective chemotherapy regimen with reduced toxicity in extensive small-cell lung cancer. *Eur. J. Cancer Clin. Oncol.*, **23**, 619–621.

MORITTU L, EARL HM, SOUHAMI RL, ASH CM, TOBIAS JS, GEDDES DM, HARPER PG AND SPIRO SG. (1989). Patients at risk of chemotherapy-associated toxicity in small cell lung cancer. *Br. J. Cancer*, **59**, 801–804.

SLEVIN ML, PLANT H, LYNCH D, DRINKWATER J AND GREGORY WM. (1988). Who should measure quality of life, the doctor or patient? *Br. J. Cancer*, **57**, 109–112.

STEPHENS RJ FOR THE BRITISH MEDICAL RESEARCH COUNCIL (MRC) LUNG CANCER WORKING PARTY (LCWP). (1994). Quality of life (QL) in randomised clinical trials: are the doctors' assessments as valid as the patients'? *Lung Cancer*, **11** (suppl. 1), 81, abstract 307.

STEPHENS RJ, GIRLING DJ, MACHIN D AND MEDICAL RESEARCH COUNCIL LUNG CANCER WORKING PARTY (1994). Treatment-related deaths in small cell lung cancer trials: can patients at risk be identified? *Lung Cancer*, **11**, 259–274.

THOMAS J STJ, LAMB D, ASHCROFT T, CORRIN B, EDWARDS CW, GIBBS AR, KENYON WE, STEPHENS RJ AND WHIMSTER WP. (1993). How reliable is the diagnosis of lung cancer using small biopsy specimens? Report of a UKCCCR Lung Cancer Working Party. *Thorax*, **48**, 1135–1139.

WORLD HEALTH ORGANIZATION (1979). *WHO Handbook for Reporting Results of Cancer Treatment*. WHO Offset Publication No. 48. WHO: Geneva.

WORLD HEALTH ORGANIZATION (1981). *International Histological Classification of Tumours No. 1: Histological Typing of Lung Tumours*, second edn. WHO: Geneva.

ZIGMOND AS AND SNAITH RR. (1983). The Hosptial Anxiety and Depression Scale. *Acta Psychiatr. Scand.*, **67**, 361–370.