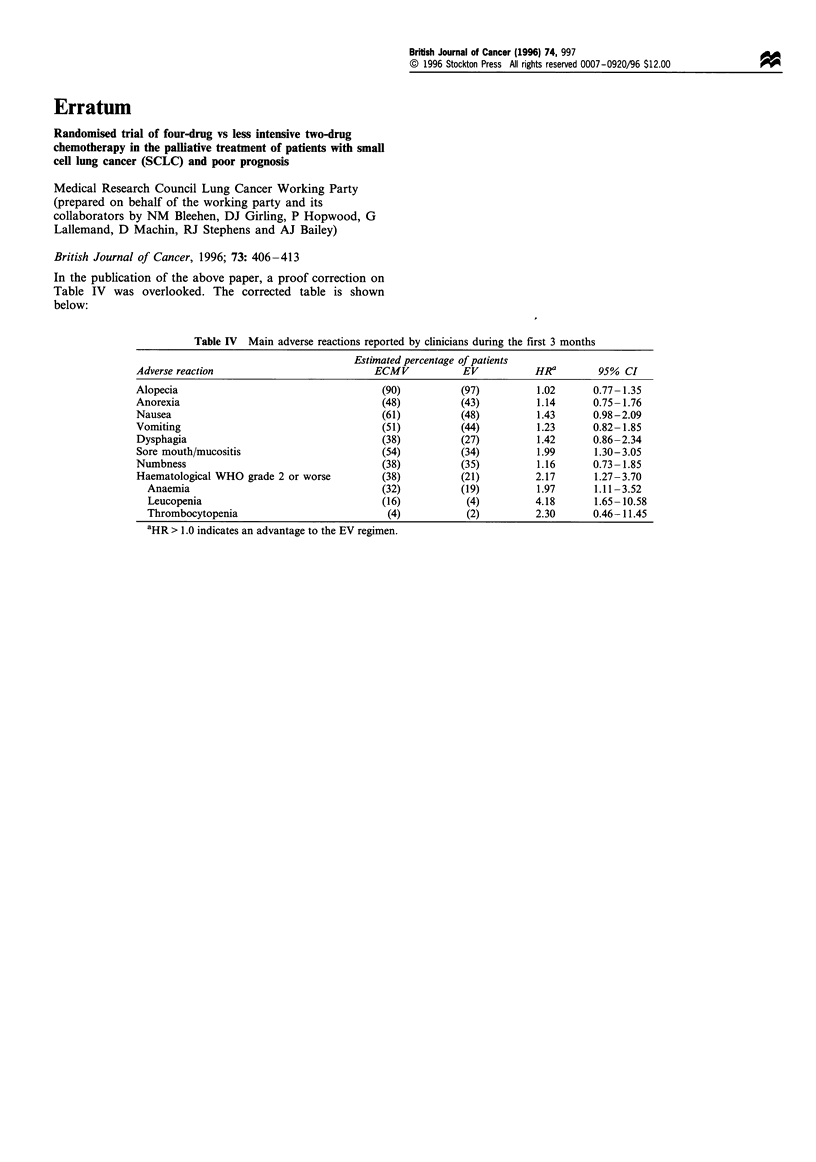# Randomised trial of four-drug vs less intensive two-drug chemotherapy in the palliative treatment of patients with small cell lung cancer (SCLC) and poor prognosis

**Published:** 1996-09

**Authors:** 


**British Journal of Cancer (1996) 74, 997**
© 1996 Stockton Press All rights reserved 0007-0920/96 $12.00

### Erratum

**Randomised trial of four-drug vs less intensive two-drug chemotherapy in the palliative treatment of patients with small cell lung cancer (SCLC) and poor prognosis**

Medical Research Council Lung Cancer Working Party (prepared on behalf of the working party and its collaborators by NM Bleehen, DJ Girling, P Hopwood, G Lallemand, D Machin, RJ Stephens and AJ Bailey)

*British Journal of Cancer*, 1996; **73:** 406-413

In the publication of the above paper, a proof correction on Table IV was overlooked. The corrected table is shown below:

**Table IV** Main adverse reactions reported by clinicians during the first 3 months

| *Adverse reaction* | *Estimated percentage of patients* ECMV | EV | *HR*[a] | *95% CI* |
|---|---|---|---|---|
| Alopecia | (90) | (97) | 1.02 | 0.77-1.35 |
| Anorexia | (48) | (43) | 1.14 | 0.75-1.76 |
| Nausea | (61) | (48) | 1.43 | 0.98-2.09 |
| Vomiting | (51) | (44) | 1.23 | 0.82-1.85 |
| Dysphagia | (38) | (27) | 1.42 | 0.86-2.34 |
| Sore mouth/mucositis | (54) | (34) | 1.99 | 1.30-3.05 |
| Numbness | (38) | (35) | 1.16 | 0.73-1.85 |
| Haematological WHO grade 2 or worse | (38) | (21) | 2.17 | 1.27-3.70 |
| Anaemia | (32) | (19) | 1.97 | 1.11-3.52 |
| Leucopenia | (16) | (4) | 4.18 | 1.65-10.58 |
| Thrombocytopenia | (4) | (2) | 2.30 | 0.46-11.45 |

[a]HR > 1.0 indicates an advantage to the EV regimen.